# Potential of mRNA-based vaccines for the control of tick-borne pathogens in one health perspective

**DOI:** 10.3389/fimmu.2024.1384442

**Published:** 2024-06-14

**Authors:** Elizabeth González-Cueto, José de la Fuente, César López-Camacho

**Affiliations:** ^1^ Peter Medawar Building for Pathogen Research, University of Oxford, Oxford, United Kingdom; ^2^ SaBio, Instituto de Investigación en Recursos Cinegéticos (IREC)-CSIC-UCLM-JCCM, Ciudad Real, Spain; ^3^ Department of Veterinary Pathobiology, Center for Veterinary Health Sciences, Oklahoma State University, Stillwater, OK, United States; ^4^ The Jenner Institute, University of Oxford, Oxford, United Kingdom

**Keywords:** one health, mRNA vaccines, cross-species immunity, tick-borne diseases, zoonotic transmission, vaccine development

## Abstract

The One Health approach, which integrates the health of humans, animals, plants, and ecosystems at various levels, is crucial for addressing interconnected health threats. This is complemented by the advent of mRNA vaccines, which have revolutionized disease prevention. They offer broad-spectrum effectiveness and can be rapidly customized to target specific pathogens. Their utility extends beyond human medicine, showing potential in veterinary practices to control diseases and reduce the risk of zoonotic transmissions. This review place mRNA vaccines and One Health in the context of tick-borne diseases. The potential of these vaccines to confer cross-species immunity is significant, potentially disrupting zoonotic disease transmission cycles and protecting the health of both humans and animals, while reducing tick populations, infestations and circulation of pathogens. The development and application of mRNA vaccines for tick and tick-borne pathogens represent a comprehensive strategy in global health, fostering a healthier ecosystem for all species in our interconnected world.

## 1 Introduction

One Health is an integrated and collaborative approach operating at local, regional, national, and global levels to improve public health outcomes. It recognizes the interconnectedness of humans, animals (both domestic and wild), plants, and ecosystems, aiming to address health threats at their interface. Emphasized by organizations like the World Health Organization (WHO) and the Centers for Disease Control and Prevention (CDC), One Health is a multisectoral, transdisciplinary strategy that achieves optimal health outcomes through collaboration and acknowledges the interconnection of various elements. It seeks to sustainably balance and optimize the health of people, animals, and ecosystems, mobilizing different sectors, disciplines, and communities to control health and ecosystem threats while addressing collective needs (1, 2). Rooted in principles of equity, socio-political parity, socioecological equilibrium, stewardship, and trans-disciplinarity, One Health integrates sectors, and perspectives to promote the well-being of all living beings and the environment in the face of present and future challenges (3).

The advent of mRNA vaccines has opened up new possibilities within this framework, offering unprecedented opportunities for disease prevention. mRNA vaccines exhibit several distinct advantages over traditional vaccine platforms, primarily due to their mechanism of action and rapid manufacturability. Unlike other vaccine platforms, mRNA vaccines leverage synthetic nucleic acid technology to instruct cells to produce a pathogen’s antigen directly, initiating a strong immunological response. This method bypasses the need for cultured cells or pathogens, substantially reducing production time and potential biosafety concerns. Furthermore, the synthetic nature of mRNA vaccines facilitates precise antigen targeting and quick adaptation to genetic drifts in pathogens, crucial for rapid response to emerging infectious diseases. Additionally, mRNA formulations can be adjusted without the extensive downstream processing required for protein vaccines, enhancing their adaptability and scalability. These attributes make mRNA vaccines a powerful tool in the rapid development of prophylactics against fast-evolving infectious agents.

The differentiation between first and second-generation mRNA vaccines is pivotal in understanding their evolution and enhanced capabilities. First-generation mRNA vaccines, like those developed for COVID-19, primarily rely on unmodified mRNA encapsulated within lipid nanoparticles (LNPs) to produce viral antigens *in situ*. These vaccines are designed to mimic natural infection, prompting the immune system to respond and develop immunity. However, they sometimes face challenges such as innate immunogenicity and rapid degradation, which can limit their efficacy and duration of protection.

Second-generation mRNA vaccines represent a significant advancement, incorporating modified nucleosides that evade innate immune detection, thereby reducing inflammation and increasing the stability of the mRNA. This modification not only enhances the translation efficiency of the mRNA but also extends the duration of antigen expression within the body, potentially improving the immunological memory. Additionally, these second-generation vaccines often employ advanced LNP designs that optimize delivery and further stabilize the mRNA, enabling targeted delivery to specific cell types or tissues. These improvements aim to increase their safety profile, reduce side effects, and expand their utility to a broader range of diseases beyond acute infectious diseases, including chronic infections, cancers, and autoimmune diseases.

## 2 Comprehensive vaccination strategies: integration and development

The critical need for comprehensive vaccination solutions demands a multifaceted approach: developing broad-spectrum, highly effective vaccines, expediting vaccine production through innovative methods, and fostering collaboration among academia, industry, and government. Moreover, the evolution of next-generation vaccines should expand beyond mRNA technology. It is imperative to create vaccine platforms that are accessible, cost-effective, and user-friendly, with practical considerations for storage, transport, and administration. Emphasizing multivalent and combination vaccine strategies and targeting systemic immune responses could be key in eliciting functional immunity and durable immunological memory (4).

The COVID-19 pandemic highlighted the transformative impact of mRNA vaccines in immunization practices. These vaccines, utilizing genetic technology to generate specific immune responses, have been pivotal in saving over 20 million lives worldwide from SARS-CoV-2 infection. mRNA and other genetic-based platforms prompt host cells to produce targeted antigens, effectively combatting a broad spectrum of infectious pathogens. The development of disease-specific mRNA vaccines, such as those targeting the spike protein of SARS-CoV-2, haemagglutinin of influenza, prM-E protein of Zika virus, and surface glycoproteins of HIV, Ebola, and rabies, illustrates the versatility and impact of this technology (5–8). Additionally, targeting non-surface antigens in complex parasites like Plasmodium (e.g., PMIF, PfGARP) or surface antigens such as the circunsporozoite protein (CSP), offer promising avenues for addressing difficult-to-treat infections (5, 9, 10). Enhanced vaccine efficacy not only protects against infectious diseases but also reduces disease incidence and severity, benefiting both human and animal populations. Thus, mRNA vaccines are poised to play a crucial role in maintaining a balanced and resilient ecosystem.

## 3 Cross-species protection

The success of inactivated and live-attenuated vaccines has significantly improved livestock productivity, promoted food security, and mitigated the morbidity and mortality associated with numerous human, animal, and zoonotic diseases. However, these conventional vaccine technologies may exhibit suboptimal efficacy against specific pathogens, and safety concerns arise with live-attenuated vaccines. Furthermore, the escalating prevalence of emerging infectious diseases highlights the urgency for the rapid deployment of new vaccines, a task that first-generation vaccines may encounter difficulties to meet (11).

Initially, there was skepticism about mRNA vaccines due to the inherent instability of RNA. Nonetheless, technological advancements in stabilizing and delivering RNA with novel Lipid Nanoparticles (LNPs) across cell membranes have paved the way for innovative vaccine designs. mRNA vaccines are able to induce CD4^+^ and CD8^+^ cellular immunity with a Th-1-type bias, although the magnitude of these cellular responses may require booster doses. This knowledge contributes to informed decision-making regarding the future implementation of mRNA vaccine platforms, including their potential in veterinary vaccinology (12, 13). The Sequivity IAV-S NA vaccine, currently the only RNA vaccine approved for animal use, specifically targets swine flu in pigs, addressing strains H1N1, H1N2, and H3N2 (14, 15).

Its development marks an advancement in veterinary immunizations but warrants a cautious interpretation regarding its broader impact on One Health—a concept that intertwines the wellness of humans, animals, and the environment. This perspective is vital, given the potential of influenza viruses to cross species barriers, potentially leading to emerging infectious diseases in humans. While this vaccine represents a step forward in managing influenza viruses within swine populations, thereby aiming to lower the risk of zoonotic transmissions, the comprehensive effects anticipated by One Health have not been fully demonstrated. The concern of the limited duration of immunity of this vaccine, a known issue with mRNA vaccines, is critical here as well (14, 15). The necessity for multiple booster vaccinations may affect many of the proposed One Health benefits, including the reduction of viral mutations and the avoidance of spillover events. Additionally, while early administration in pigs supports the economic health of swine herds and addresses antibiotic resistance by potentially reducing secondary infections, these aspects are part of a broader conversation about sustainable practices and the mitigation of environmental impacts. This narrative underlines the importance of further research and interdisciplinary collaboration to genuinely harness the benefits of such vaccines within the One Health framework, ensuring they contribute meaningly to the health of animals, humans, and our shared environment.

mRNA vaccines possess a remarkable attribute: their ability to provide antigen expression that could encode cross-species specific vaccines. Customizing the mRNA to target specific pathogen(s) across animal and human vaccines will enable adaptation for various animal species. In addition to murine models, potency studies in pigs and cattle, for example, may have greater applicability and benefit to humans. Moreover, techniques such as biolistics devices for pig skin delivery offer insights for intradermal delivery in humans and vice versa (16). Furthermore, the existence of shared or similar pathogens, such as influenza viruses, other viruses, parasites, and mycobacteria, among human and animal populations underscores the importance of vaccination strategies. Utilizing vaccines, including mRNA vaccines as well as inactivated or vectored vaccines, in animal populations could interrupt the transmission cycles of zoonotic pathogens. This approach is not exclusive to mRNA vaccine technology but applies broadly to various vaccine platforms, all of which can significantly lower the risk of disease outbreaks and enhance the health security of both humans and animals (17).

## 4 Disease surveillance, early warning and vaccine deployment:

Monitoring the infection and vaccination status of animal populations provides crucial insights into disease dynamics, enabling proactive measures to prevent the spread of infections. Such surveillance not only protects animal health but also serves as an early warning mechanism for potential spillover events into human populations. Complementing disease surveillance, early warning systems play a vital role in the timely detection of infectious disease outbreaks. These systems, which integrate data from various sources such as healthcare facilities, laboratories, and digital health platforms, are instrumental in quickly identifying potential threats (18). In the realm of mRNA vaccines, the agility of these early warning systems becomes even more significant. They enable the rapid identification of new pathogen strains, thereby facilitating the swift adaptation of mRNA vaccines to these emerging threats. This rapid adaptability of mRNA technology underscores its value, providing a flexible and responsive tool in the global arsenal against infectious diseases. The deployment of mRNA vaccines in controlling infectious diseases is a multi-faceted process characterized by several critical stages. A key advantage of mRNA vaccines lies in their rapid production capability (19). While mRNA vaccines have the advantage of rapid design and production following the identification of a pathogen’s genetic sequence, this benefit is somewhat specific and depends on the nature of the pathogen. By expressing specific subunit antigens, mRNA vaccines necessitate detailed knowledge of protective antigens that can elicit an immune response. This requirement can limit their rapid-response capability in dealing with complex or yet-to-be-characterized pathogens, such as many bacteria or certain viruses, where identifying a single protective antigen may not be straightforward. In these instances, vaccine technologies that introduce whole pathogens, whether inactivated or attenuated, might offer a more effective immediate response due to their comprehensive antigenic presentation. Despite this, the need for efficient distribution and logistics remains critical for mRNA vaccines, particularly because of their cold storage requirements, which demand a robust cold chain system to maintain vaccine efficacy during transport and storage. Developing effective vaccination strategies is another pivotal aspect, requiring considerations such as identifying target populations, appropriate timing, and dosage requirements. This is particularly important as public health authorities prioritize vaccination for vulnerable groups and high transmission areas. Equally important are public education and training campaigns (20). Given the novel nature of mRNA technology, addressing vaccine hesitancy and misinformation is as crucial as training healthcare workers in the specifics of mRNA vaccine storage, handling, and administration.

Post-vaccination surveillance is crucial to monitor vaccine efficacy, track breakthrough infections, and identify any side effects. This continuous monitoring is critical in a global context, necessitating international collaboration to coordinate vaccine production, distribution, and ensure equitable access, especially in low- and middle-income countries. Genomic surveillance plays a key role in rapidly detecting changes that necessitate adjustments in mRNA vaccine production and adaptation. The flexibility of mRNA vaccines to be quickly updated in response to evolving conditions highlights their critical role in addressing global health challenges, making them a dynamic and responsive tool in the fight against infectious diseases (21).

## 5 Environmental impact

The introduction of mRNA vaccines presents not only a breakthrough in human and animal health but also a potential tool for addressing environmental challenges. These vaccines, by curbing the spread of infectious diseases, can substantially reduce the reliance on antimicrobial agents. This reduction is crucial in veterinary and human medicine, as it can help to slow down the development of antimicrobial resistance (AMR), a growing concern in global health, something that is yet to be investigated (22, 23). The environmental benefits of these vaccines are in line with One Health principles. However, it is essential to consider the environmental footprint of these vaccines, encompassing the manufacturing, usage, and disposal phases, as well as the carbon emissions associated with transportation. The production process of mRNA vaccines involves multiple steps that could have environmental implications, such as using raw materials and energy consumption. Additionally, the use phase includes considerations related to cold chain logistics, which require energy-intensive refrigeration systems. The disposal of vaccine-related materials, such as syringes and vials, also poses environmental challenges. Furthermore, the distribution of vaccines globally involves transportation, which contributes to carbon emissions. To truly align with the One Health approach, a comprehensive strategy is needed that not only maximizes the health benefits of mRNA vaccines but also minimizes their environmental impacts. This requires a balanced assessment of the vaccines’ entire lifecycle, from production to disposal, ensuring sustainable practices are integrated at every step. Efforts to optimize the production process for greater efficiency, improve waste management strategies, and enhance distribution logistics to reduce carbon emissions are vital components of this strategy (24).

## 6 Integrative strategies in controlling tick-borne diseases: a one health approach to vaccination and tick management

An interesting strategy that has been pursued since decades ago is the development of anti-tick vaccines (25, 26). This approach is based on the premise that one organism could be immunized with an antigen that belongs to the actual tick (i.e., tick saliva components or tick midgut components). Such antigen would elicit an immune response that will recognize the components of the tick that is in the process of feeding from the vaccinated organism. The establishment of such responses will lead to anti-tick antibodies to hinder the process of tick feeding, either by eliciting tick detachment or by inducing an inflammatory immune response. This response is crucial as it is directed not against a pathogen carried by the tick but against the tick itself. The goal is to prevent the tick from successfully feeding and, as a result, decrease the chance of transmitting pathogens to the host as well as decrease tick populations, which would help to control the prevalence of tick-borne diseases.

Significant progress has been made in the discovery of tick antigens and the creation of vaccines targeting these antigens. These vaccines, which utilize tick-derived antigens from saliva (27, 28) or midgut, aim to interrupt the tick’s feeding cycle, thereby blocking the spread of tick-borne diseases. Among the developed subunit vaccines, Gavac^®^ and TickGARD^®^ based on BM86 antigen from the tick’s midgut, aim to disrupt the tick’s feeding and reproduction processes. Of them, only Gavac^®^ is now available in the market. These vaccines have been significant in controlling cattle tick infestations. Gavac^®^, within integrated tick management systems, has shown effectiveness in reducing acaricidal applications needed to control certain *Rhipicephalus microplus* cattle tick strains. However, its efficacy is limited against other tick species.

Anti-tick vaccine approaches hold several advantages. Firstly, it reduces the likelihood of transmission of various tick-borne diseases, such as Lyme disease, by targeting the common vector. Secondly, it offers a more holistic method of disease prevention, aligning with the One Health approach by considering the ecological role of ticks and the broader environmental implications of disease transmission. Moreover, anti-tick vaccines could potentially reduce the reliance on chemical acaricides, which are commonly used to control tick populations but pose environmental risks and can lead to the development of acaricide-resistant tick populations.

In 2021, a notable study demonstrated the potential of nucleoside-modified mRNA-LNP vaccines in tick immunity, specifically examining the efficacy of a salp14 mRNA-LNP vaccine in guinea pigs. This vaccine outperformed salp14 DNA or protein immunizations in terms of the speed of immune response. Notably, the mRNA-LNP vaccine induced pronounced erythema at the tick bite site within 18 hours post-immunization, compared to the slower response (24 hours or more) observed with other immunization methods. This rapid onset of erythema is a critical indicator of a robust immune reaction, suggesting that the mRNA-LNP platform might be more effective in eliciting both humoral and cellular immune responses. Erythema, as a marker of tick vaccine efficacy, signifies active immune cell engagement at the bite site. This response not only facilitates early tick detection and removal but also plays a crucial role in reducing pathogen transmission. The findings of this study highlight the potential advantages of mRNA-LNP vaccines in enhancing immune responses against tick bites. Moreover, it opens avenues for further research and development in the field of tick vaccine efficacy, underscoring the innovative applications of mRNA vaccine technologies (29).

Moreover, a newly developed OspA-encoding mRNA-LNP vaccine against *Borrelia burgdorferi* has shown superior immune responses in mice when compared to alum-adjuvanted OspA protein subunit vaccinated mice (30). Rapid pre-clinical testing and evaluation of these vaccines could lead to a reduction in tick populations and lower transmission rates of tick-borne diseases.

Recent advances in vaccine development for controlling ticks and tick-borne pathogens have focused on mRNA vaccines (31). An innovative aspect of this strategy is the potential vaccination of wildlife reservoirs, which play a key role in the propagation of pathogens between ticks. Vaccinating wildlife reservoirs with mRNA vaccines presents unique challenges, apart from the actual costs, together with the suitability of other administration routes such as oral vaccination. The stability of lipid nanoparticle (LNP)-encapsulated RNA and cold chain requirements for mRNA vaccines pose significant logistical challenges in wild-life settings, where maintaining optimal storage conditions is difficult. In this regard, thermostable LNPs and novel mRNA-LNP formulations are now under developing to address such challenges. Additionally, the current production costs of mRNA vaccines are relatively high, potentially limiting large-scale deployment in wildlife populations. The necessity for booster doses further complicates this approach, as re-vaccinating wild animals at consistent intervals may not be feasible. Despite these hurdles, this approach not only offers a more sustainable and effective method of disease control but also aligns with environmental conservation objectives by reducing the dependence on chemical acaricides. Oral vaccine delivery platforms are also key to target wildlife species, but these are mainly based for subunit-based vaccines (32); whether mRNA-based vaccines could be used for such purpose remains to be explored in the future. Additionally, combining these vaccines with innovative approaches like quantum vaccinomics and para-transgenesis target multiple tick vector species and pathogen genetic variants (33).

**Figure 1:**
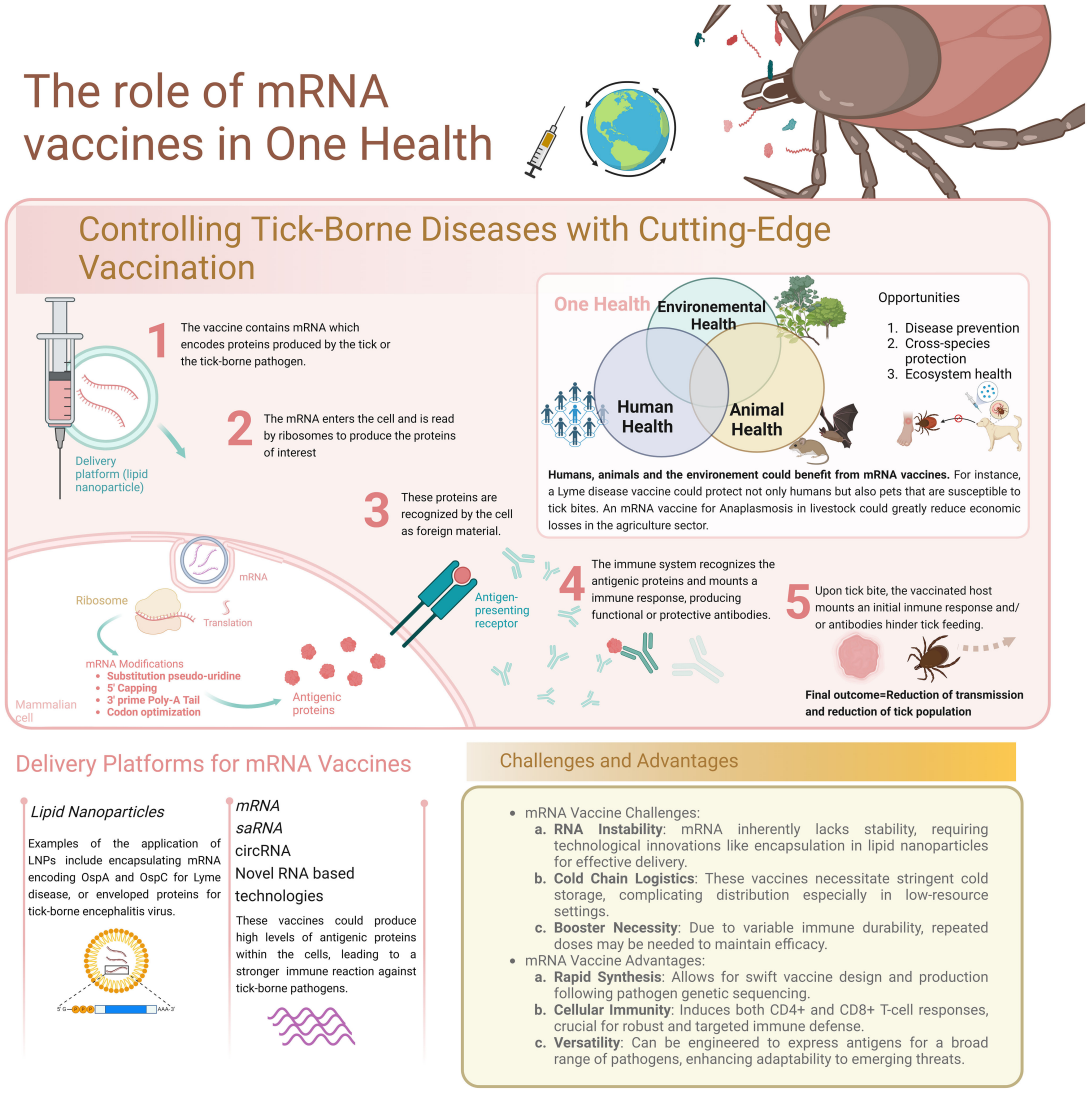
The role of mRNA vaccines in One Health.

In the context of tick-borne diseases, mRNA vaccines excel over traditional platforms due to their rapid configurability and synthesis. Utilizing lipid nanoparticle (LNP)-encapsulated mRNA, these vaccines directly program host cells to produce relevant antigens, thereby bypassing the slower processes of culturing and inactivating pathogens. This swift production cycle is pivotal for responding to emergent and mutating tick-borne pathogens, ensuring timely vaccine updates and deployment (**Figure 1**).

## 7 mRNA vaccines combatting bacterial pathogens transmitted by ticks

### 7.1 Lyme disease

Lyme disease, caused by the bacterium *Borrelia burgdorferi*, is a significant public health concern and one of the most prevalent tick-borne illnesses, particularly in North America and Europe. Transmitted to humans through the bite of infected *Ixodes* ticks, commonly known as black-legged or deer ticks, Lyme disease presents a variety of symptoms, ranging from fever, headache, fatigue, and a characteristic skin rash known as erythema migrans. If left untreated, the infection can spread to the joints, heart, and nervous system, leading to more severe health complications. The prevalence of Lyme disease has been increasing, with numerous factors contributing to its spread, including changes in land use, climate change, and increased human-tick interactions. According to the Centers for Disease Control and Prevention (CDC), it is estimated that 476,000 people might be diagnosed with the disease of Lyme disease in the United States each year (34, 35).

Currently, the protein subunit vaccine VLA15, targeting Lyme disease, is in Phase 3 human trials (VALOR) (NCT05477524) (36). This development is significant, as there has been no Lyme disease vaccine available for human use since the withdrawal of LYMErix in 2002 (37, 38). However mRNA vaccines provide a promising solution for combatting Lyme disease. Targeting *Borrelia burgdorferi* antigens like OspA or OspC could protect both humans and animals from lyme disease (30, 39). In this context, the development of the 19ISP mRNA vaccine, containing lipid nanoparticles encoding 19 *I. scapularis* salivary proteins, is particularly groundbreaking (40). This vaccine boosts the host’s immunity to tick bites, reducing tick feeding and *Borrelia burgdorferi* transmission. Positive findings in guinea pig models show reduced tick engorgement and hindered pathogen transmission. The 19ISP vaccine’s ability to induce an early immune response and disrupt the feeding process of *I. scapularis* ticks presents a dual benefit: it not only impedes tick engorgement but also prevents the transmission of Lyme disease and other tick-borne infections. This innovative approach, potentially used alone or in an anti-tick/anti-*Borrelia* combinatorial based vaccines expressed in single mRNA platform, could represent a significant advancement in Lyme disease control and prevention. This aligns with the One Health approach, addressing the intricate interplay between humans, animals, and the environment. Additionally, emphasizing the importance of early tick removal further reduces the risk of infection. This holistic strategy is crucial for effectively tackling the growing challenge of Lyme disease and other vector-borne diseases.

### 7.2 Anaplasmosis and ehrlichiosis

Anaplasmosis and Ehrlichiosis are increasingly recognized as important emerging infectious diseases, they are tick-borne diseases that pose significant health risks to both humans and animals (41, 42). Anaplasmosis is primarily caused by *Anaplasma phagocytophilum*, which is responsible for Human Granulocytic Anaplasmosis (HGA). This disease is known for its acute febrile symptoms, including fever, headache, and muscle aches, and in some cases, can lead to more severe health complications if not promptly treated (43). Ehrlichiosis, often caused by *Ehrlichia chaffeensis*, presents similar acute symptoms and also poses a serious health risk. Human granulocytic anaplasmosis is of particular concern due to its ability to infect humans directly through tick bites, making it a critical public health issue in areas with high tick activity (41). The development of mRNA vaccines offers a promising avenue for controlling these diseases, this technology holds distinct advantages over traditional protein or DNA vaccine approaches. By designing vaccines that target key antigens specific to *A. phagocytophilum* and *E. chaffeensis*, it is possible to induce a robust immune response in the vaccinated organism (44, 45). This feature is especially beneficial for vaccinating animals, such as livestock and companion animals, which serve as reservoirs for these diseases. Specifically targeting *A. phagocytophilum* and E. chaffeensis antigens through mRNA vaccines could offer a significant tool in preventing disease transmission to humans by effectively reducing the pathogen load in these animal hosts.

### 7.3 Rickettsial diseases

Tick-borne rickettsial diseases, such as Rocky Mountain Spotted Fever, pose significant health risks to both humans and animals. These diseases are caused by various *Rickettsia* species, with Rocky Mountain Spotted Fever being caused by *Rickettsia rickettsii* (46). The advent of mRNA vaccines offers a promising solution to tackle such diseases. These vaccines can be specifically tailored to target key antigens of *Rickettsia* species, such as outer membrane protein A (OmpA) or the surface cell antigen (Sca) (47, 48). This targeted approach holds great potential for providing cross-species protection, significantly reducing the burden of these diseases. It’s crucial to note that while a few antigens from *Rickettsia* species have been identified for vaccine development, they represent only a small portion of the extensive array of proteins these pathogens produce. This observation underscores the need for further exploration in the realms of vaccinology and bacteriology to fully understand and utilize the potential of these antigens in developing effective vaccines against rickettsial diseases.

## 8 mRNA vaccines combatting viral agents transmitted by ticks

### 8.1 Tick-borne encephalitis (TBE) virus

Tick-borne encephalitis (TBE) is a viral infection transmitted by ticks, primarily found in parts of Europe, Russia, and Asia, and can lead to severe neurological complications in humans, including meningitis, encephalitis, and meningoencephalitis. The severity of these complications underscores the need for effective preventative measures, particularly in areas where the TBEV is endemic. Using mRNA technology, vaccines can induce an immune response against specific TBE virus antigens like envelope glycoprotein E or pre-membrane protein prM. Notably, TICOVAC, an approved vaccine in the USA and Europe, offers higher immunogenicity than previous TBE vaccines (49–51). TICOVAC is a purified, inactivated virus vaccine, what sets this vaccine apart from its predecessors is its higher immunogenicity, which translates to a stronger and more effective immune response in individuals vaccinated against TBE (49, 50, 52).

While the current inactivated vaccines for Tick-Borne Encephalitis Virus (TBEV) have a well-established safety and efficacy profile, mRNA vaccines present several compelling advantages that may complement or enhance TBEV immunization strategies. Firstly, mRNA vaccines can be rapidly developed and scaled, which is advantageous for responding to emergent strains or outbreaks. This agility in vaccine design and production allows for precise targeting of immunogenic viral proteins, such as the Envelope glycoprotein, and potentially improving immunological outcomes in specific populations, such as the immunocompromised or elderly, who may benefit from a more focused immune response. Moreover, mRNA vaccines do not contain live virus, eliminating the risk of vaccine-induced disease, and can be formulated with lipid nanoparticles that enhance immune responses without additional adjuvants. However, the stringent cold chain requirements for mRNA vaccines pose logistical challenges, particularly in low- and middle-income countries. Nonetheless, thermostable Lipid Nano Particles could tackle this problem in the next generation of mRNA-based vaccines. Given these considerations, while inactivated vaccines continue to be reliable, mRNA vaccines could offer strategic benefits in scenarios requiring rapid deployment and high adaptability.

### 8.2 Powassan virus

The Powassan virus is an emerging and serious tick-borne flavivirus prevalent in North America and parts of Russia. It is known for causing severe neurological complications in humans, including life-threatening encephalitis. The virus is transmitted primarily through tick bites, making it a significant public health concern in regions where these ticks are endemic (53). Despite its growing impact, there are currently no vaccines or specific treatments available for Powassan virus infection, underlining the urgency for effective medical countermeasures. The development of mRNA vaccines targeting specific antigens of the Powassan virus represents a promising approach to prevent and control this emerging infectious disease. By utilizing mRNA vaccine platforms, targeted vaccines can be developed against specific Powassan virus antigens, such as the envelope glycoprotein (E) or the non-structural protein (NS1), to prevent infection in both human and animal populations. Currently, a live-attenuated chimeric vaccine candidate is under development, with promising results (54). In addition, research has shown promising developments in the use of mRNA vaccines against the Powassan virus. A study highlighted the development of a lipid nanoparticle (LNP)-encapsulated modified mRNA vaccine encoding the Powassan virus prM and E genes. This vaccine demonstrated immunogenicity and efficacy in mice, inducing high titers of neutralizing antibody and providing sterilizing immunity against different Powassan virus strains. Additionally, the vaccine induced cross-neutralizing antibodies against multiple other tick-borne flaviviruses and protected mice against the distantly related Langat virus (55). This demonstrates the potential of LNP-mRNA vaccine platforms in developing vaccines for protection against multiple flaviviruses, including Powassan virus and further provides evidence that vaccines against these diseases are able to fit into a One-Health approach.

### 8.3 Crimean Congo hemorrhagic fever

Crimean-Congo Hemorrhagic Fever (CCHF) is a severe tick-borne disease caused by the CCHF virus (CCHFV) and it is endemic in Africa, the Balkans, the Middle East, and Asian countries south of the 50th parallel north, posing a significant public health challenge and with high mortality rate (56, 57). The One Health approach is crucial for addressing CCHF, which involves integrated strategies such as vaccine development, environmental management, and tick control. To this end, recent advancements in vaccine technology have opened new avenues for CCHF prevention. The use of mRNA vaccines could be a valuable strategy to complement other Crimean-Congo Hemorrhagic Fever (CCHF) vaccine platforms, such as the adenoviral vectored ChAdOx1 vaccine. Such platform, which gained prominence with the Oxford-AstraZeneca COVID-19 vaccine, delivers genetic material from the CCHFV to elicit a specific immune response (58). While anti-vector immunity is a notable limitation of adenoviral vector vaccines in shorter intervals between immunisations, anti-vector immune response declines long-term, allowing for effective subsequent vaccinations with the same vector. Nonetheless, a strategic advantage of mRNA vaccines, which do not induce anti-vector immunity could be either used in heterologous vaccination approaches, or as a suitable alternative platform to tackle CCHF.

Notably, mRNA vaccine platforms have shown promising results. Research has demonstrated that nucleoside-modified mRNA vaccines, using lipid nanoparticles to deliver the genetic material of CCHFV, can protect against the virus. These vaccines, targeting the virus’s nucleoprotein (N) or glycoproteins (GcGn), have been effective in inducing robust immune responses in animal models (59). Moreover, research has demonstrated that alphavirus-based replicating RNA (repRNA) vaccines, expressing viral nucleoprotein (NP) and glycoprotein precursor (GPC), provide robust protection against lethal CCHFV challenges in animal models, primarily through non-neutralizing anti-NP antibodies and GPC-specific T-cell responses (60).

A novel self-replicating RNA vaccine using a Venezuelan Equine Encephalitis Virus (VEEV) replicon and a cationic nanocarrier has shown promising outcomes. This vaccine targets either the CCHFV nucleoprotein (NP) or glycoprotein precursor (GPC). Despite the challenges with glycoproteins’ variability, the NP vaccine generated strong, non-neutralizing antibodies and fully protected mice against a highly divergent strain of CCHFV. On the other hand, the GPC vaccine mainly triggered a CD8 T-cell response but did not protect alone. Combining NP and GPC vaccines, however, significantly enhanced viral control (61).

The development of vaccines against CCHF aligns with the One Health approach by potentially reducing the transmission risk from animal reservoirs to humans and mitigating the disease’s impact on public health. Additionally, understanding and controlling tick vectors in the environment play a critical role in reducing the incidence of CCHF, further emphasizing the importance of a holistic approach to disease management.

## 9 Conclusion

The integration of One Health principles with the advancements in mRNA vaccine technology leads to a transformative era in public health. This synergy addresses the complex nature of infectious diseases across human, animal, and environmental health. One Health’s holistic approach, which merges human, veterinary, and environmental sciences, is crucial in combating health threats at their confluence. The success of mRNA vaccines during the COVID-19 pandemic has revolutionized immunization practices. These vaccines stand out for their rapid development and customization capabilities, making them key players in responding to emerging health threats. Beyond human medicine, their application in veterinary care is pivotal in controlling animal diseases and reducing zoonotic transmission risks. In the fight against tick-borne diseases such as Lyme disease, Anaplasmosis, Ehrlichiosis, and Tick-Borne Encephalitis, mRNA vaccines represent a promising solution. They present significant advantages for immunocompromised individuals and the elderly by inducing potent immune responses, which are crucial due to the reduced immunogenicity these groups experience with conventional vaccines. The adaptability of mRNA technology also allows for the rapid development of vaccines that can target multiple pathogens simultaneously, providing comprehensive protection in regions burdened by diverse tick-borne diseases. Their ability to target specific pathogens provide a flexible and effective approach to these growing health concerns. Furthermore, One Health emphasizes environmental sustainability in health strategies. mRNA vaccines would contribute to the reduction reliance on antimicrobials, thus contributing to the fight against antimicrobial resistance. They also enhance disease surveillance and early warning systems, allowing for proactive responses to emerging pathogens. The potential of mRNA vaccines to confer cross-species immunity disrupts zoonotic disease transmission cycles, protecting both human and animal health. The alignment of these vaccines with One Health principles underscores a strategic approach to global health challenges, paving the way for a more resilient ecosystem and a healthier future for all species.

## Data availability statement

The original contributions presented in the study are included in the article/supplementary material. Further inquiries can be directed to the corresponding author.

## Author contributions

EG-C: Writing – review & editing, Writing – original draft, Visualization. JD: Writing – review & editing, Writing – original draft. CL-C: Writing – review & editing, Writing – original draft, Supervision, Funding acquisition, Conceptualization.
